# CAFs-derived SCUBE1 promotes malignancy and stemness through the Shh/Gli1 pathway in hepatocellular carcinoma

**DOI:** 10.1186/s12967-022-03689-w

**Published:** 2022-11-08

**Authors:** Jungang Zhao, Rizhao Li, Jiacheng Li, Ziyan Chen, Zixia Lin, Baofu Zhang, Liming Deng, Gang Chen, Yi Wang

**Affiliations:** 1 Department of Hepatobiliary Surgery, The First Affiliated Hospital of Wenzhou Medical University, 325000 Wenzhou, Zhejiang China; 2 Key Laboratory of Diagnosis and Treatment of Severe Hepato-Pancreatic Diseases of Zhejiang Province, The First Affiliated Hospital of Wenzhou Medical University, Wenzhou, China; 3 Department of Epidemiology and Biostatistics, School of Public Health and Management, Wenzhou Medical University, 325000 Wenzhou, Zhejiang China

**Keywords:** Hepatocellular carcinoma, SCUBE1, Tumour microenvironment, Stemness

## Abstract

**Background:**

The tumour microenvironment and cirrhotic liver are excellent sources of cancer-associated fibroblasts (CAFs), which participate in carcinogenesis. Thus, it is important to clarify the crosstalk between CAFs and HCC cells and the related mechanism in regulating carcinogenesis.

**Methods:**

Human hepatocellular carcinoma (HCC) tissues and matched adjacent normal tissues were obtained from HCC patients. Immunohistochemistry, Western blotting (WB) and RT–qPCR were performed to detect the expression of SCUBE1. The roles of SCUBE1 in inducing stemness features in HCC cells were explored and investigated in vitro and in vivo. Student’s t tests or Mann–Whitney U tests were used to compare continuous variables, while chi-square tests or Fisher’s exact tests were used to compare categorical variables between two groups.

**Results:**

SCUBE1 was confirmed to be highly expressed in CAFs in HCC and had a strong connection with stemness and a poor prognosis. In addition, CAFs were found to secrete SCUBE1 to enhance the malignancy of HCC cells and increase the proportion of CD133-positive cells. Silencing SCUBE1 expression had the opposite effect. The Shh pathway was activated by SCUBE1 stimulation. Inhibition of cyclopamine partially reversed the stimulating effect of SCUBE1 both in vivo and in vitro. Moreover, based on the RT–qPCR, ELISA and WB results, a high SCUBE1 expression level was found in HCC tissue and serum.

**Conclusion:**

This study revealed that CAFs-derived SCUBE1 can enhance the malignancy and stemness of HCC cells through the Shh pathway. This study aims to provide new perspectives for future HCC studies and provide new strategies for HCC treatment.

**Supplementary Information:**

The online version contains supplementary material available at 10.1186/s12967-022-03689-w.

## Introduction

Hepatocellular carcinoma (HCC) is the most common histological type of primary liver cancer, and has a low five-year survival rate of less than 20%.[1] Hepatitis virus infection, alcohol use, NAFLD and other factors have been verified to be causes of HCC.[2] Although there are curative treatment options for HCC including surgery, liver transplantation, transarterial chemoembolization (TACE) and targeted molecular therapy, it is still difficult to control the progression to advanced liver cancer.[3, 4] Thus, the development of new therapies for liver cancer is crucial and urgent.

The tumour microenvironment (TME) comprises various types of cells and stromal components.[5] The TME has been confirmed to play an important role in promoting angiogenesis, immune escape and vascular invasion. As the most abundant cells in the TME, CAFs are a crucial source of extracellular matrix and can promote the production of interstitial connective tissue.[6, 7] CAFs can also accelerate proliferation, migration, invasion, and epithelial-mesenchymal transition (EMT) and drug resistance in HCC.[8, 9] Since most liver fibrosis and cirrhosis cases eventually develop into liver cancer, these liver tissues are usually enriched in fibroblasts, which are differentiated from resting fibroblasts and hepatic stellate cells.[8, 10–12] In addition, by regulating the chemical attraction of extracellular matrix, fibrosis, and other interstitial cells, CAFs can enhance the connection between liver tumours and the interstitium.[6, 7] Crosstalk between HCC cells and CAFs facilitates the possibility of tumour immune escape.[13] Blocking this crosstalk can significantly inhibit the promotive effect. [14]Therefore, in-depth experiments are needed to better understand the role of CAFs in HCC.

SCUBE1, a secreted glycoprotein anchored on the cell membrane, plays an important role in bone development and the occurrence of vascular diseases. [15] SCUBE1 has been verified to regulate tumorigenesis capacity in different cancer types. SCUBE1 can bind to the BMP domain to inhibit tumour growth, and the serum content of SCUBE1 in gastric cancer patients is significantly higher than that in healthy people.[16, 17] Emerging studies have reported a pivotal role of the SCUBE family as an important upstream regulator in regulating the secretion of Sonic Hedgehog (Shh) protein. [18, 19] The Shh pathway is involved in the acquisition of stem cell properties in tumours. [20, 21] Yan et al. found that endothelial cells can secrete Shh protein to activate the Shh/Gli1 pathway in adjacent melanoma cells, thereby obtaining a stemness phenotype. [22] Therefore, SCUBE1 seems to be correlated the acquisition of stemness features by tumour cells.

In this study, we demonstrated the role of SCUBE1 in promoting the occurrence of HCC and explored whether SCUBE1 participates in the acquisition of stem cell-like self-renewal attributes through the Shh pathway. In addition, SCUBE1 was found to be a promising, clinically useful target gene, and its clinical significance for diagnosis and targeted treatment approaches deserves in-depth study.

## Materials and methods

### Patient samples and cell culture

The tumour tissues and adjacent tissues, paraffin sections and serum used in the experiments were obtained from The First Affiliated Hospital of Wenzhou Medical University with the approval of the ethics committee of the First Affiliated Hospital of Wenzhou Medical University.

Two hepatocellular carcinoma cell lines (PLC/PRF/5 and Huh7) and the human hepatic stellate cell line LX2 were obtained from the Cell Bank of the Chinese Academy of Sciences (Shanghai, China). HCC cell lines and LX2 cells were cultured in DMEM (Gibco, United States) supplemented with 10% foetal bovine serum (Gibco, USA), 100µg/mL streptomycin and 100 U/mL penicillin (Sigma, USA) at 37°C with 5% CO_2_.

### Immunoblotting assay and coimmunoprecipitation

Pretreated cells were lysed with RIPA buffer containing protease inhibitor cocktail (Roche, USA) and phenylmethylsulfonyl fluoride (PMSF) on ice. Then, lysates were quantified with a BCA assay (Thermo Fisher Scientific, USA). Then, equal amounts of protein from each sample were separated via 10% sodium dodecyl sulfate–polyacrylamide gel electrophoresis and transferred to PVDF membranes. After blocking with 5% skimmed milk for 2h at room temperature, the membranes were incubated with primary antibodies against either SCUBE1 (Abcam, ab101358, 1:1000), Shh (Abcam, ab53281, 1:1000), Smo (Abcam, ab236465, 1:1000), Gli1 (Abcam, ab134906, 1:1000), CD133 (CST, 64326s, 1:1000), Vimentin (CST, 5741, 1:1000), α-SMA (Abcam, 5694, 1:1000), Nanog (Abcam, ab109250, 1:1000), Sox2 (Abcam, ab92494, 1:1000) or β-actin (Abcam, ab8226, 1:1000) at 4°C overnight. The membranes were soaked in horseradish peroxidase (HRP)-conjugated secondary antibody for an hour the next day. Protein bands were visualized with SuperSignal West Femto Maximum Sensitivity Substrate (Thermo Scientific, Waltham, MA, USA).

After washing with PBST, the magnetic beads were incubated with anti-Shh (Santa Cruz, sc-373779) and IgG (Santa Cruz, sc-2025) antibodies for 1h. Then, the beads were incubated with lysates overnight at 4°C. After supernatant removal, the beads were washed with PBST and incubated in diluted elution buffer at 70°C for 10min. Finally, the protein complexes were analysed by SDS‒PAGE/immunoblot analysis.

### RNA extraction and RT‒qPCR

Total RNA was extracted from cells or frozen tissues using TRIzol reagent (Life Technologies, USA) and reverse-transcribed into single-stranded cDNA with a PrimeScript™ RT Reagent Kit (Takara, Japan). Quantitative real-time polymerase chain reaction was performed on an ABI PRISM 7500 real-time PCR system to quantify mRNA expression levels. The relative expression levels were calculated using the 2^−ΔΔCt^ method after normalization to β-actin. The primers used for RT–qPCR are listed in Table1.

**Table 1:** Primer sequences used for RT-qPCR analysis

Gene	Forward primer (5’-3’)	Reverse primer (5’-3’)
**CD133**	**GTGGCGTGTGCGGCTATGAC**	**CCAACTCCAACCATGAGGAAGACG**
**SCUBE1**	**GAGGATGAGTGCGGCGATGTTC**	**CTCTCGTAGGTCTGGCAGGTCTC**
**Nanog**	**CAATGGTGTGACGCAGGGAT**	**TGCACCAGGTCTGAGTGTTC**
**Sox2**	**GCCGAGTGGAAACTTTTGTCG**	**GGCAGCGTGTACTTATCCTTCT**
**β-actin**	**CACGATGGAGGGGCCGGACTCATC**	**TAAAGACCTCTATGCCAACACAGT**

### Cell viability assay

Pretreated cells were trypsinized and seeded in a 96-well plate at a density of 3000 cells per well. Then, the cells were treated with CCK-8 reagent (Dojindo, Japan) at 37°C for 2h, and the OD values were measured at 450nm with a microplate reader (BioTek, Winooski, VT, USA) for five consecutive days.

### Isolation and purification of CAFs

Tumour tissues and adjacent tissues were washed with PBS three times and cut into small pieces, followed by digestion with collagenase IV. Then, the suspension was centrifuged and filtered through a 40-µm cell strainer. The supernatants were centrifuged, washed and incubated in DMEM with high glucose medium containing 10% foetal serum to obtain fibroblasts. The expression levels of α-SMA and vimentin were measured to identify CAFs.

### Coculture system

CAFs or activated LX2 cells were plated in the upper chamber of a Transwell chamber with a 0.4-µm pore size at a density of 1 × 10^5^/ml, and HCC cells were seeded in the lower chamber. After cocultivation for 24h, the HCC cells were used for other experiments.

### Migration assay

Cell migration was assessed using Transwell chambers with 8μm-pores (Corning Inc., Corning, NY, USA). A total of 5 × 10^4^ cells suspended in 100µl of serum-free DMEM were seeded into the upper chamber, and 600µl DMEM containing 20% FBS was used as the chemotactic agent in the lower chamber. After 24h, nonmigrated cells were removed from the upper surface of the Transwell using a cotton swab. Cells on the Transwell membranes were fixed in methanol for 20min, dried, and then stained with crystal violet for 30min. Cells that migrated through the cell membrane to the lower surface were photographed via microscopy and counted using ImageJ.

### Colony formation assay

Different treated cells were inoculated into six-well plates at a density of 1000 cells per well. The medium was changed every three days. After 14 days, the plates were washed with PBS and fixed with paraformaldehyde for 30min, followed by 0.1% crystal violet staining for 30min. The number of colonies with more than 50 cells was counted by ImageJ. The experiment was repeated three times.

### Immunofluorescence staining

Cells were cultured on glass coverslips overnight and fixed with 4% paraformaldehyde for 20min. Fixed cells were stained with CD133 (CST, 64326s, 1:200), vimentin (CST, 5741, 1:200), and α-SMA (Abcam, 5694, 1:200) antibodies, followed by Alexa Fluor-conjugated secondary antibody, and nuclei were counterstained with diaminophenyl indole (DAPI). Representative images were acquired via a fluorescence microscope Leica DMi8 with Leica LAS X software and analyzed with ImageJ.

### Transfection assay

To regulate the expression level of SCUBE1, we used Lipo3000 to transiently transfect CAFs with knockdown plasmid or overexpression plasmid according to the manufacturer’s instructions. The transfected cells were then used in subsequent experiments. The plasmids were purchased from Genechem (Shanghai, China).

### Animal studies

To evaluate the effect of CAFs on the tumorigenesis of HCC cells in vivo, 18 BALB/c nude mice were divided into three groups. HCC cells alone or together with CAFs at a ratio of 8:1 or together with CAFs and cyclopamine were suspended in 100 µL of PBS and subcutaneously implanted into the right flanks of nude mice. Cyclopamine (25mg/kg body weight) was intraperitoneally injected into mice each day when the subcutaneous tumour volume reached 200 mm^3^. Two weeks later, the mice were sacrificed. The subcutaneous tumours were frozen and partly fixed in paraformaldehyde.

### RNA sequencing (RNA-seq)

Total RNA of LX2 and activated LX2 cells was extracted using TRIzol reagent (Invitrogen) and shipped to BGI (Shenzhen, China) for further RNA-seq detection and analysis using the BGISEQ‐500 sequencer. mRNAs with a fold change > 2, FDR < 0.05 and p value < 0.05 between different groups were selected as significantly differentially expressed genes (DEGs). GO enrichment and KEGG enrichment analyses were applied to DEGs.

The sequencing data has been submitted to national center for biotechnology information (NCBI) Sequence Read Archive (SRA, http://www.ncbi.nlm.nih.gov/bioproject/) under the accession number PRJNA855034.

### Immunohistochemical staining

Paraffin-embedded tissues were cut into 4-µm sections. Then, tissue sections were dewaxed in xylene and hydrated in different concentrations of ethanol. After antigen retrieval, H_2_O_2_ was added to block endogenous peroxidase, and 5% BSA was applied to block nonspecific staining for 1h. Primary antibodies against Ki-67 (Abcam, 16667, 1:200) and SCUBE1 (SigmaAldrich, HPA003190, 1:30) were added and incubated at 4°C overnight. The next day, the sections were incubated with horseradish peroxidase-labelled secondary antibodies for 1h at 37°C. Afterwards, DAB and haematoxylin were used for staining, and the stained sections were photographed with a microscope. In each experiment, negative controls were established with PBS instead of primary antibody. Ki-67 positive staining was quantified by ImageJ software.

### Multiplex immunohistochemistry (mIHC)

FFPE tissue sections were dewaxed and hydrated. Then, sections were blocked by H_2_O_2_ for 10min and BSA for an hour in order after antigen retrieval. Then, the sections were incubated with primary antibodies at 4°C and the secondary antibodies were added the next day. Then, sections were subjected to multi-color immunohistochemistry with Four-color multiplex fluorescent immunohistochemical staining kit (Absin, abs50028, China) following the manufacturer’s instructions. Primary antibodies are as follows: vimentin (CST, 5741, 1:200), α-SMA (Abcam, 5694, 1:200) and SCUBE1 (SigmaAldrich, HPA003190, 1:30). Microscopy images were photographed using Leica Thunder system (Leica, Wetzlar, Germany).

### Enzyme-linked immunosorbent assay (ELISA)

The cell supernatant and patient serum were collected. After that, ELISA kits were used to detect the content of SCUBE1 (CUSABIO, CSB-E15005h) or Shh (CUSABIO, CSB-E12005h) according to the manufacturer’s procedure.

### Statistical analysis

All the data are presented as the mean ± standard deviation (SD) of three independent experiments. Student’s t test was used to compare two independent samples, and one-way analysis of variance (ANOVA) was used to compare multiple groups. The log-rank (Mantel‒Cox) test was performed for the comparison of Kaplan‒Meier survival curves. GraphPad Prism 8.0 and SPSS 22.0 software were used for statistical analyses. *P* < 0.05 was considered to indicate a statistically significant difference.

## Results

**1. The stemness and malignancy of HCC cells were significantly improved after coculture with LX2 cells**.

**Fig. 1:**
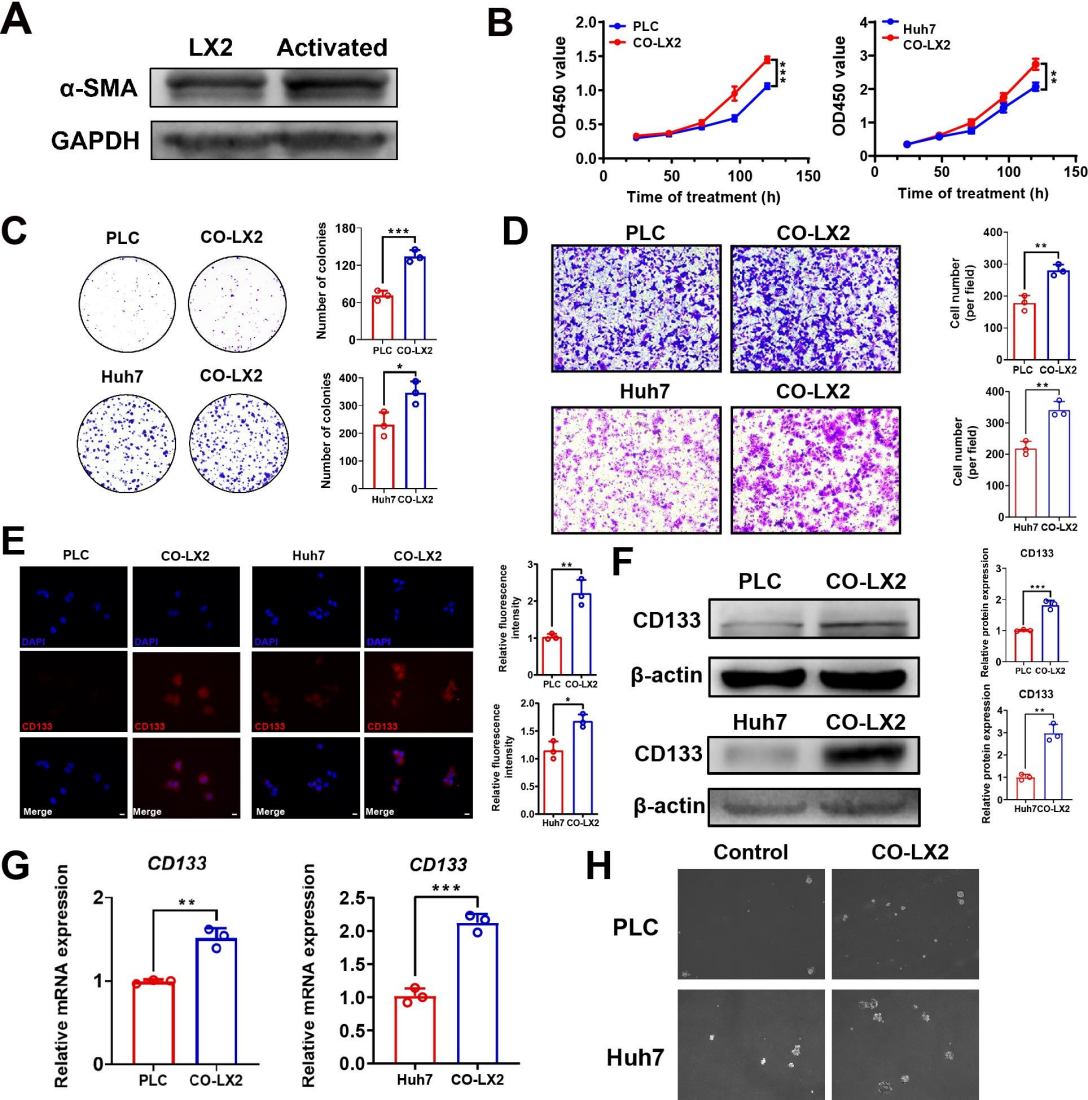
**HCC malignancy was promoted after cocultivation.(A)** The expression of α-SMA in LX2 cells and cocultured LX2 cells was detected by WB. **(B)** Cell viability was evaluated with CCK-8 assays of PLC and Huh7 cells cocultured with LX2 cells (**P < 0.01, ***P < 0.001, t test). **(C)** The colony formation ability of PLC and Huh7 cells cocultured with activated LX2 cells was detected with colony formation assays (*P < 0.05, ***P < 0.001, t test). **(D)** The migration ability of HCC cells and cocultured HCC cells was compared using Transwell assays (**P < 0.01, t test). Scale bar, 200μm **(E)** Immunofluorescence was used to assess CD133 protein expression in HCC cells and cocultured HCC cells (*P < 0.05, **P < 0.01, t test). Scale bar, 10μm. CD133 fluorescence density values in HCC cells were quantified before and after coculture. **(F)** Western blotting was used to assess CD133 protein expression in HCC cells and cocultured HCC cells (**P < 0.01, ***P < 0.001, t test). **(G)***CD133* mRNA expression was detected by RT–qPCR (**P < 0.01, ***P < 0.001, t test). **(H)** Sphere formation assays were used to evaluate the self-renewal capacity of treated and untreated HCC cells. Scale bar, 500μm. Data are represented as mean ± SD of three independent experiments

Hepatic stellate cells can be activated after coculture with human HCC cells. Activated liver stellate cells and liver cancer fibroblasts have similar properties in promoting tumorigenesis. To determine whether LX2 cells could be activated after coculture with hepatoma cells, WB was used to detect α-SMA expression in LX2 cells. The results showed that the expression of α-SMA in LX2 cells was significantly increased after cocultivation (Fig.1A). To clarify the effect of activated LX2 cells on HCC cells, LX2 cells were grown in cocultures with HCC cells. The proliferation of PLC and Huh7 cells in cocultures tested with CCK-8 assays was significantly improved (Fig.1B). Activated LX2 cells also significantly enhanced the migration and colony formation ability of HCC cells (Fig.1C-D). These results indicated that the malignant capacity of HCC cells could be improved by coculture with activated hepatic stellate cells. Schlegel reported that conditioned medium extracted from mouse fibroblasts increased the expression of stemness genes, including Sox2, Oct4, Nanog, and Klf4, in liver cancer cells. [23, 24] According to these findings, we performed immunofluorescence to detect whether activated HSCs could promote the expression of stem cell-associated genes in HCC cells. The results revealed that cocultured HCC cells had higher CD133 fluorescence intensity than untreated HCC cells (Fig.1E). Then, the expression of CD133 was detected by WB and RT–qPCR. We found that cocultured PLC and HCC cells had higher CD133 protein content and transcription levels (Fig.1F-G). Sphere formation assays also confirmed that the self-renewal capacity of PLC and Huh7 cells was significantly improved after stimulation with activated LX2 cells (Fig.1H). These results revealed that the stemness and malignancy of HCC cells can be significantly enhanced after coculture with LX2 cells.

### 2. SCUBE1 is highly expressed and secreted in CAFs

**Fig. 2:**
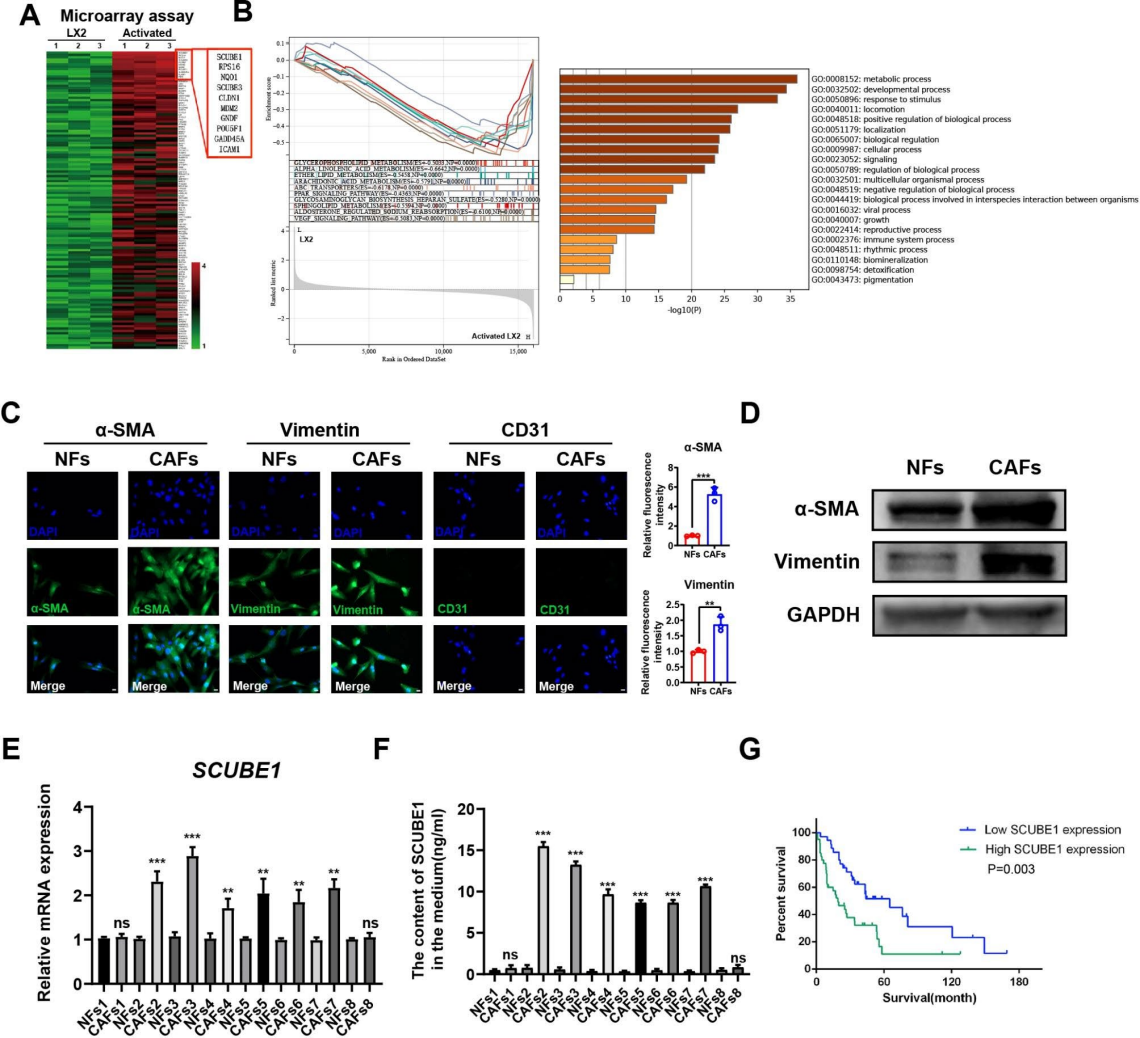
**SCUBE1 was highly expressed in CAFs and associated with stemness.(A)** Gene changes in LX2 cells and cocultured LX2 cells were detected by transcriptome sequencing. **(B)** KEGG pathway enrichment analysis and GO functional annotation were used. **(C)** α-SMA, vimentin and CD31 expression in CAFs and NFs was detected by immunofluorescence staining (**P < 0.01, ***P < 0.001, t test). Scale bar, 10μm. **(D)** Western blotting was used to detect the mRNA expression of *SCUBE1* in different CAFs and NFs. **(E)** RT–qPCR was used to detect the mRNA expression of *SCUBE1* in different CAFs and corresponding NFs (**P < 0.01, ***P < 0.001, t test). **(F)** The secretion of SCUBE1 in the supernatants of different NFs and CAFs was detected by ELISAs (***P < 0.001, t test). **(G)** Kaplan–Meier analysis was performed to compare the low SCUBE1 expression group and the high SCUBE1 expression group. Data are represented as mean ± SD of three independent experiments

To clarify how activated LX2 cells promote the proliferation, migration, and stemness of HCC cells, activated and untreated LX2 cells were collected for transcriptome sequencing to detect changes in gene expression. Multiple upregulated and downregulated genes are listed in Table2. Based on the sequencing results, SCUBE1 was selected among several significantly upregulated genes in the cocultured group (Fig.2A). Predicted molecular functions were assigned to genes identified using gene ontology (GO) functional annotation and KEGG pathway enrichment analysis (Fig.2B). Besides, Tumor Immune single-cell Hub (TISCH), a single-cell sequencing database, showed that SCUBE1 expression was higher in CAFs than that in cancer cells in most cancers (**Supplementary Fig.1**). After that, we extracted CAFs and normal fibroblasts (NFs) from 8 fresh HCC tissues and adjacent tissues. Alpha smooth muscle actin (α-SMA) and vimentin are specific markers of CAFs. The expression levels of α-SMA and vimentin were higher in CAFs than in normal fibroblasts, and the expression of CD31 was negative according to the immunofluorescence staining results (Fig.2C), which verified that the extracted cells were fibroblasts rather than endothelial cells. WB showed that the protein expression levels of α-SMA and vimentin were higher in CAFs than in NFs (Fig.2D). RT–qPCR was then used to detect SCUBE1 mRNA levels in CAFs. Six out of eight CAF groups showed higher SCUBE1 mRNA expression levels than NFs (Fig.2E). To further verify the secretion of SCUBE1 in the coculture system, we cocultured CAFs and NFs with HCC cells, extracted the supernatant, and detected the secretion of SCUBE1 protein via ELISA. The results showed that the level of SCUBE1 protein secreted by CAFs was higher than that secreted by NFs (Fig.2F). Survival analysis revealed a poor prognosis in HCC patients with high SCUBE1 expression based on the TCGA database (Fig.2G). These results suggest that CAFs generally secrete high levels of SCUBE1 protein without cell-to-cell contact, which is correlated with a poor prognosis.

**Table 2:** The top ten unregulated and downregulated genes

Upregulated genes	Downregulated genes
SCUBE1	Slc25a51
RPS16	Gng11
NQO1	Lmod3
SCUBE3	Gucy2g
CLDN1	Dusp27
MDM2	Prxl2a
GNDF	Pnpla1
POU5F1	Cd164l2
GADD45A	Fcho1
ICAM1	Tmem196

### 3. High SCUBE1 secretion by CAFs can promote the malignant progression of HCC cells

**Fig. 3:**
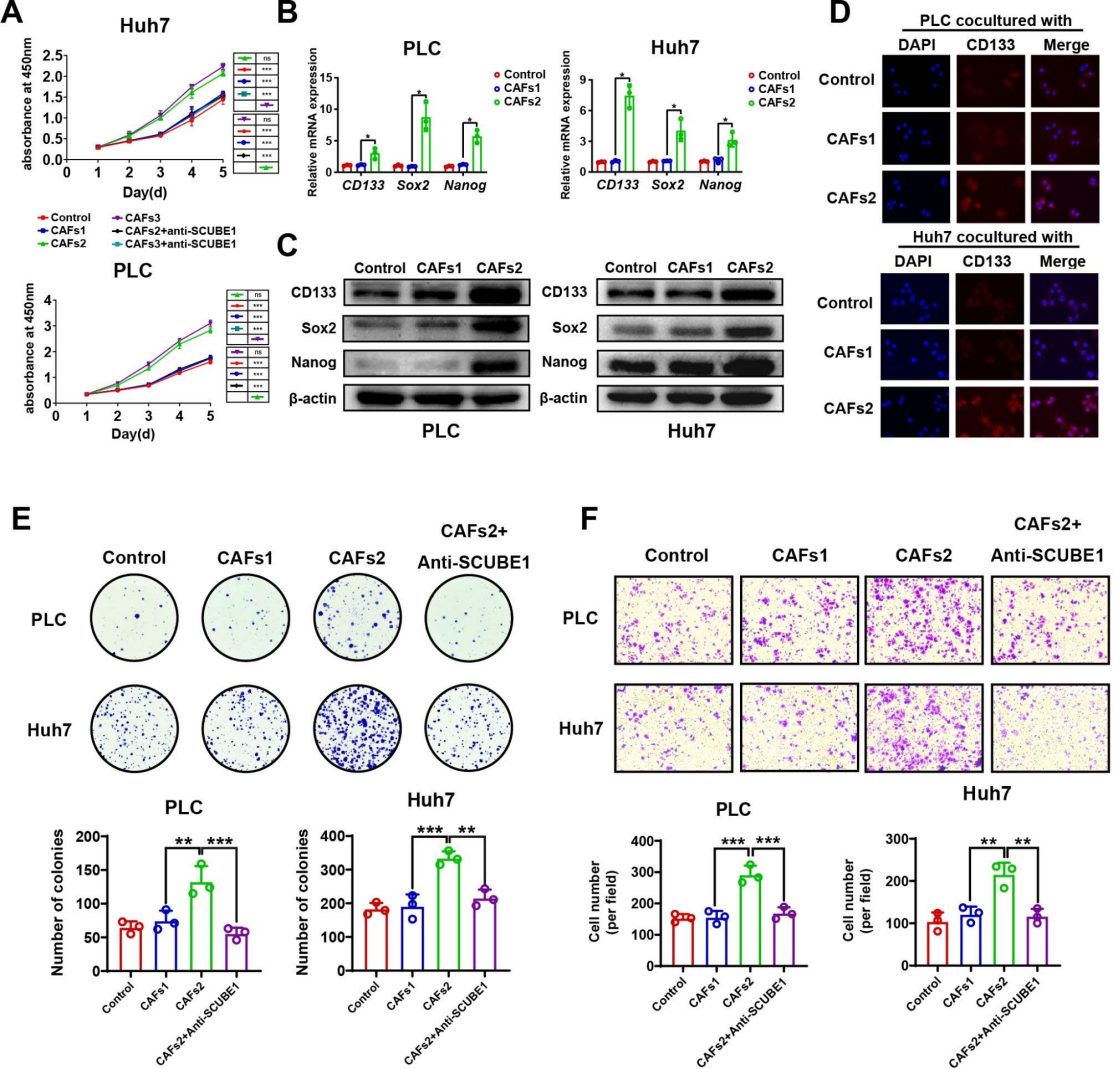
**CAFs-derived SCUBE1 can regulate the malignant progression of HCC cells.(A)** PLC and Huh7 cells were cocultured with CAFs1, CAFs2 and CAFs3 with or without anti-SCUBE1 antibody. Cell viability was measured with CCK-8 assays (***P < 0.001 vs. CAFs2 or CAFs3, one-way ANOVA). **(B)** RT–qPCR was performed to determine the mRNA levels of *CD133*, *Sox2* and *Nanog* in PLC and Huh7 cells cocultured with CAFs1 and CAFs2 (*P < 0.05, t test). **(C)** The expression levels of CD133, Sox2 and Nanog in treated and untreated HCC cells were evaluated by Western blotting (***P < 0.001, t test). **(D)** CD133 was detected via immunofluorescence staining in different treated HCC cells (**P < 0.01, t test). Scale bar, 10μm. **(E)** The effects of CAFs1, CAFs2 and anti-SCUBE1 antibody on the colony formation ability of PLC and Huh7 cells (*P < 0.05, **P < 0.01, ***P < 0.001 vs. CAFs2, one-way ANOVA). **(F)** HCC cell migration ability was evaluated with Transwell assays after coculture with CAFs1 and CAFs2 with or without an anti-SCUBE1 antibody (**P < 0.01, ***P < 0.001 vs. CAFs2, one-way ANOVA). Scale bar, 200μm. Data are represented as mean ± SD of three independent experiments

PLC and Huh7 cells were cocultured with CAFs1, CAFs2 and CAFs3, and SCUBE1 antibody was added. The results showed that compared with that of CAFs1, the proliferation ability of HCC cells was greatly improved after coculture with CAFs2 and CAFs3, but this promotion was inhibited by the addition of an anti-SCUBE1 antibody (Fig.3A). There was not much difference between CAFs2 and CAFs3 in terms of the promotive effect; therefore, CAFs1 and CAFs2 were chosen for follow-up experiments. To elucidate whether SCUBE1 can regulate the stemness and malignancy process in HCC, PLC and Huh7 cells were cocultured with CAFs1 and CAFs2, followed by extraction of total RNA and measurement of the expression levels of stemness-related genes (CD133, Nanog, and Sox2). HCC cells had higher expression of stemness-related genes after cocultivation with CAFs2 than after cocultivation with CAFs1 (Fig.3B). Similarly, WB results showed that the protein expression levels of CD133, Nanog, and SOX2 were significantly increased after coculture with CAFs2 compared with CAFs1 (Fig.3C). Immunofluorescence assays showed that PLC cells and Huh7 cells gained more CD133 expression after coculture with CAFs2 (Fig.3D). Compared with coculture with CAFs1, the colony formation ability and migration ability of PLC and Huh7 cells cocultured with CAFs2 were significantly increased, but the promotive effect was antagonized after addition of the anti-SCUBE1 antibody (Fig.3E-F). Wound healing assays showed the same trend **(Supplementary Fig.2A-B)**. Therefore, we preliminarily concluded that CAFs with high SCUBE1 expression and secretion can promote the stemness and malignancy of HCC cells.

### 4. SCUBE1 can enhance the stemness and malignancy of HCC cells through the Shh/Gli1 pathway

**Fig. 4:**
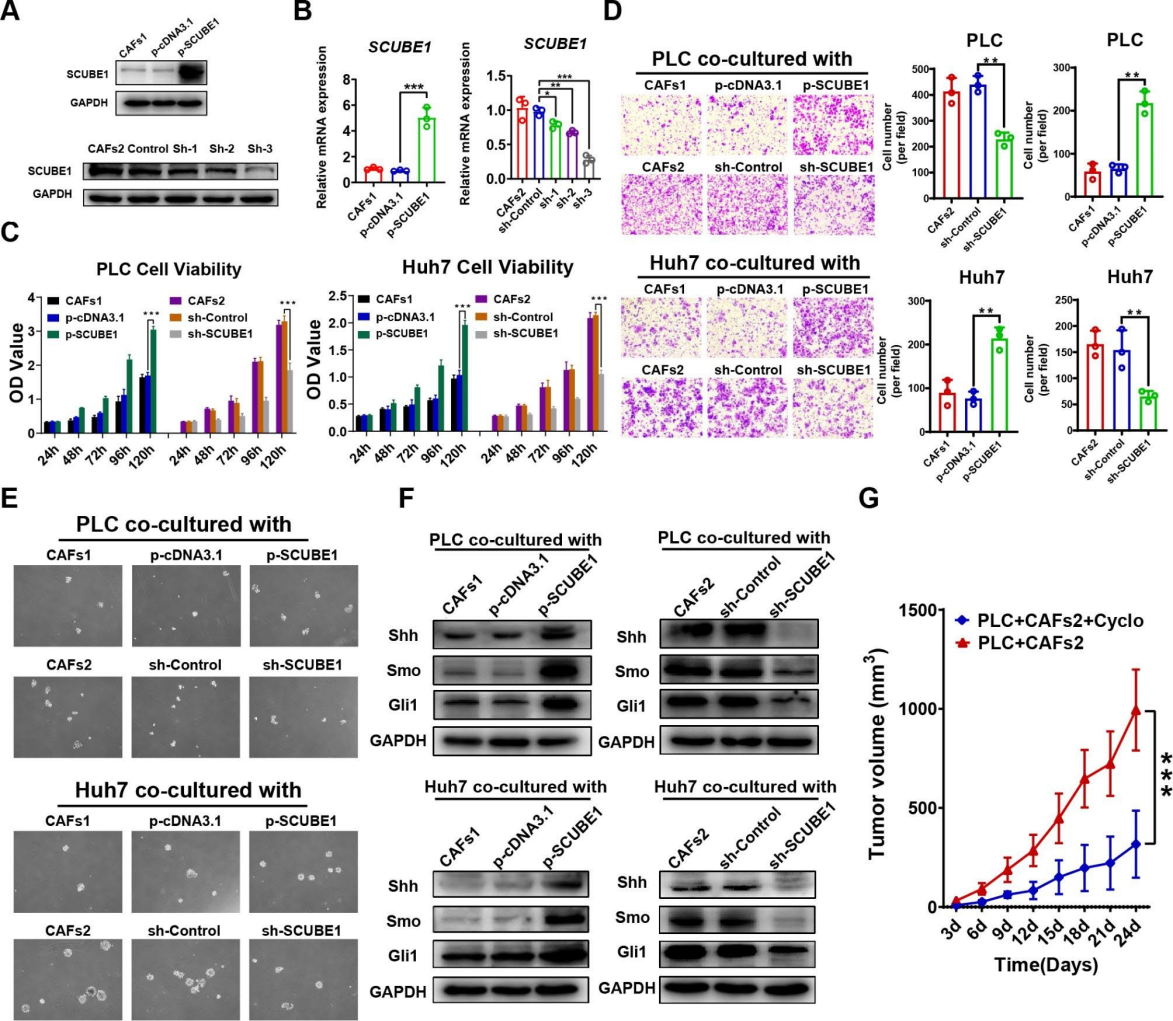
**Effects of altered SCUBE1 expression in CAFs on the malignancy of HCC cells.(A)** WB was used to detect the transfection efficiency of the overexpression plasmid and knockout plasmid. **(B)** RT–qPCR was used to detect the transfection efficiency of the overexpression plasmid (***P < 0.001 t test) and knockout plasmid (*P < 0.05, **P < 0.01, ***P < 0.001 vs. sh-Control, one-way ANOVA). **(C)** Cell viability was measured with CCK-8 assays in HCC cells subjected to different treatments (***P < 0.001, t test). **(D)** The effect of SCUBE1 silencing and overexpression in CAFs on the migration of HCC cells was measured with Transwell assays (**P < 0.01, t test). Scale bar, 200μm. **(E)** Sphere formation ability was detected in PLC cells and Huh7 cells. Scale bar, 500μm. **(F)** Shh, Smo, and Gli1 protein expression levels were detected via Western blotting in PLC and Huh7 cells cocultured with different treated CAFs. **(G)** Growth curve of mouse subcutaneous tumours derived from different treated PLC cells at the indicated time points (***P < 0.001, t test). Data are represented as mean ± SD of three independent experiments

To determine the functional significance of SCUBE1, gain- and loss-of-function studies were performed in CAFs. CAFs1 were transfected with overexpression plasmids, and CAFs2 were transfected with the knockdown plasmid. After that, the different CAF treatment groups were collected and subjected to SDS–PAGE to detect the transfection efficiency of the plasmids. WB and RT–qPCR showed that the expression levels of SCUBE1 in transfected CAFs1 were significantly higher than those in the control group, while SCUBE1 expression in sh-3 plasmid-transfected CAFs2 was significantly lower than that in the empty plasmid group (Fig.4A-B). WB showed that SCUBE1 knockdown had no effect on the expression of phenotypic markers in CAFs (**Supplementary Fig.2C**). Subsequently, PLC and Huh7 cells were cocultured with treated CAFs1 and CAFs2. The cell viability and migration ability of cocultured HCC cells were reduced after SCUBE1 expression levels were decreased in CAFs, while increasing SCUBE1 expression had a promotive effect (Fig.4C-D). Spheroid formation assays showed the same trend (Fig.4E). The above results confirmed that elevated SCUBE1 levels could indeed promote the malignancy process and stemness of HCC cells. A recent study reported that the SCUBE family is a critical factor in promoting Sonic Hedgehog (Shh) protein activity. Therefore, we extracted protein from each treatment group and detected changes in the Shh/Gli1 pathway. The results showed that increased SCUBE1 expression increased the expression levels of Shh, Smo and Gli1 and that decreased SCUBE1 expression led to a decrease in these protein expression levels (Fig.4F). After that, PLC cells were mixed with the CAFs2 and SCUBE1-knockdown groups and subcutaneously implanted into mice to construct xenograft models. When the tumour volume reached 1200 mm^3^, the mice were sacrificed, and HE staining was performed. The results showed that the tumour cell proliferation rate was significantly increased after coculture with CAFs2, but this effect disappeared when SCUBE1 expression was downregulated (Fig.4G). In conclusion, regulation of SCUBE1 expression in CAFs can affect the secretion of SCUBE1 to influence the malignant process and stemness of HCC in vivo and in vitro via the Shh/Gli1 pathway.

### 5. Blocking the shh pathway can inhibit the promotion of HCC by SCUBE1

**Fig. 5:**
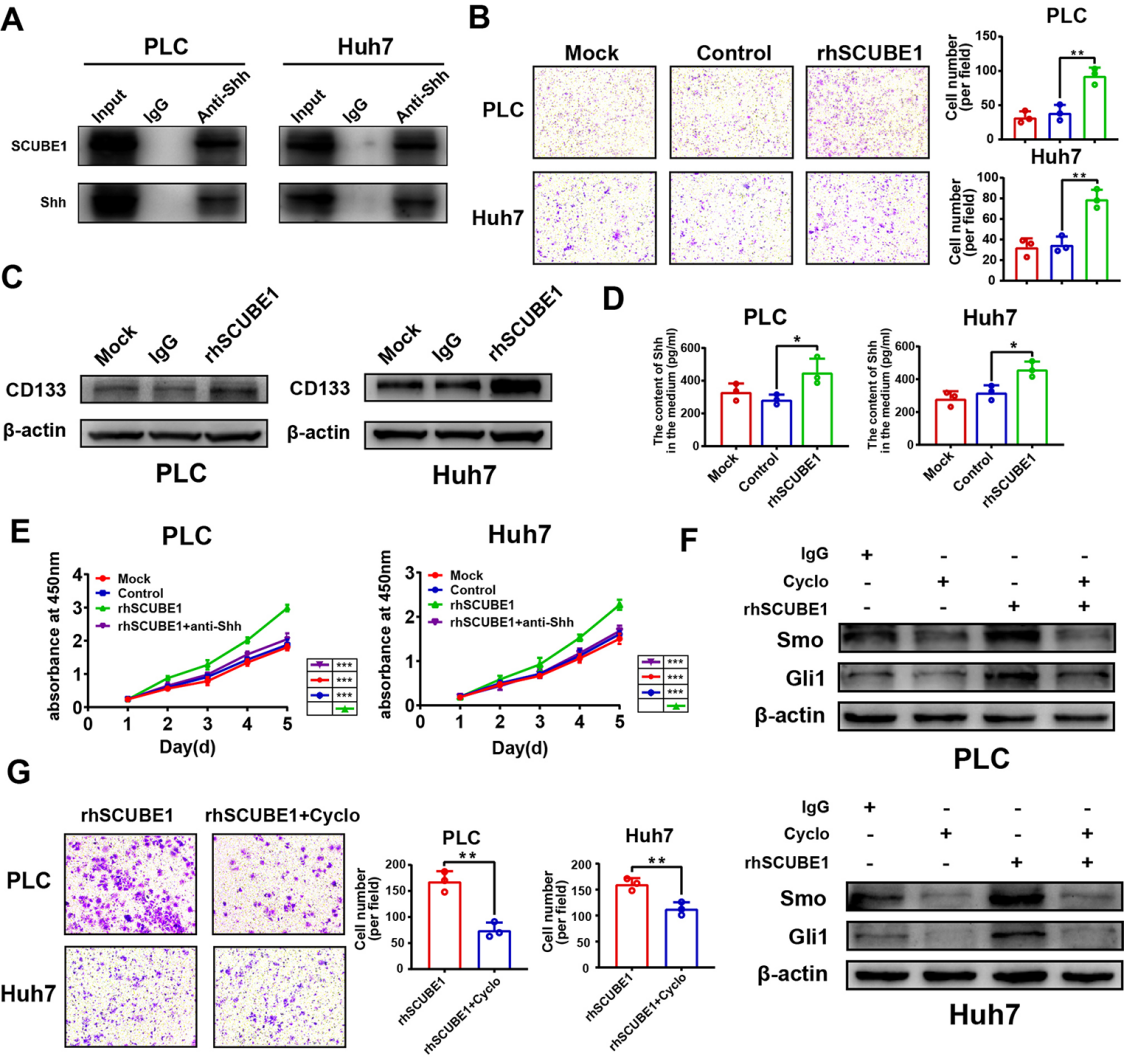
**Blocking the Shh pathway can reverse the stimulatory effect of SCUBE1.(A)** Coimmunoprecipitation of SCUBE1 and Shh in PLC and Huh7 cells. The IgG isotype was used as a negative control. **(B)** The invasion capacity of PLC and Huh7 cells treated with rhSCUBE1 and IgG was detected with Transwell assays (**P < 0.01, t test). Scale bar, 200μm. **(C)** The expression of CD133 in different groups was measured via Western blotting. **(D)** The Shh content in the supernatant in PLC and Huh7 cells with different treatments was measured with ELISA (*P < 0.05, t test). **(E)** The proliferation rates of PLC and Huh7 cells after the addition of rhSCUBE1 with or without Shh antibody were measured (***P < 0.001 vs. rhSCUBE1, one-way ANOVA). **(F)** Western blot analysis of Smo and Gli1 expression in PLC and Huh7 cells treated with rhSCUBE1 and cyclopamine. β-Actin was used as an internal control. **(G)** The invasion capacity of PLC and Huh7 cells treated with rhSCUBE1 and cyclopamine was measured with Transwell assays (**P < 0.01, t test). Scale bar, 200μm. Data are represented as mean ± SD of three independent experiments

To investigate whether there is a direct interaction between SCUBE1 and Shh, coimmunoprecipitation experiments were performed, and the interaction was demonstrated (Fig.5A). The invasion ability of HCC cells was increased by recombinant human SCUBE1 protein (rhSCUBE1) stimulation (Fig.5B). Therefore, the exogenous addition of rhSCUBE1 can enhance the malignant behaviours of PLC and Huh7 cells. To explore whether rhSCUBE1 has an effect on CD133 expression, we used WB to detect changes in CD133. The results showed that under rhSCUBE1 protein stimulation, CD133 protein expression was significantly increased (Fig.5C). To further verify the above conclusions, rhSCUBE1 and IgG protein were incubated with PLC and Huh7 cells, respectively, and the culture supernatant was collected. It was also found that after incubation with rhSCUBE1, the secretion of Shh in the supernatant significantly increased (Fig.5D). CCK-8 assay results showed that exogenous addition of SCUBE1 promoted the proliferation of HCC cells, which could be antagonized by an anti-Shh antibody (Fig.5E). WB also confirmed that exogenous addition of SCUBE1 promoted the expression of Shh pathway-related proteins, and the use of an anti-Shh antibody inhibited this effect (**Supplementary Fig.3A**). Cyclopamine is a well-known Smo inhibitor. The half-maximal inhibitory concentration (IC50) of cyclopamine was measured in PLC and Huh7 cells, and 20 µM was determined to be the optimal concentration for cell treatment (**Supplementary Fig.3B-C**). This compound inhibited Smo and Gli1 expression and inhibited the stimulatory effect of rhSCUBE1 (Fig.5F). The promotive effect of rhSCUBE1 on the invasion ability of HCC cells was also inhibited by cyclopamine (Fig.5G). Thus, we concluded that SCUBE1 can promote Shh expression and secretion and enhance the malignant properties of HCC through the Shh pathway.

### 6. SCUBE1 promotes tumorigenesis in vivo through the Shh/Gli1 pathway

**Fig. 6:**
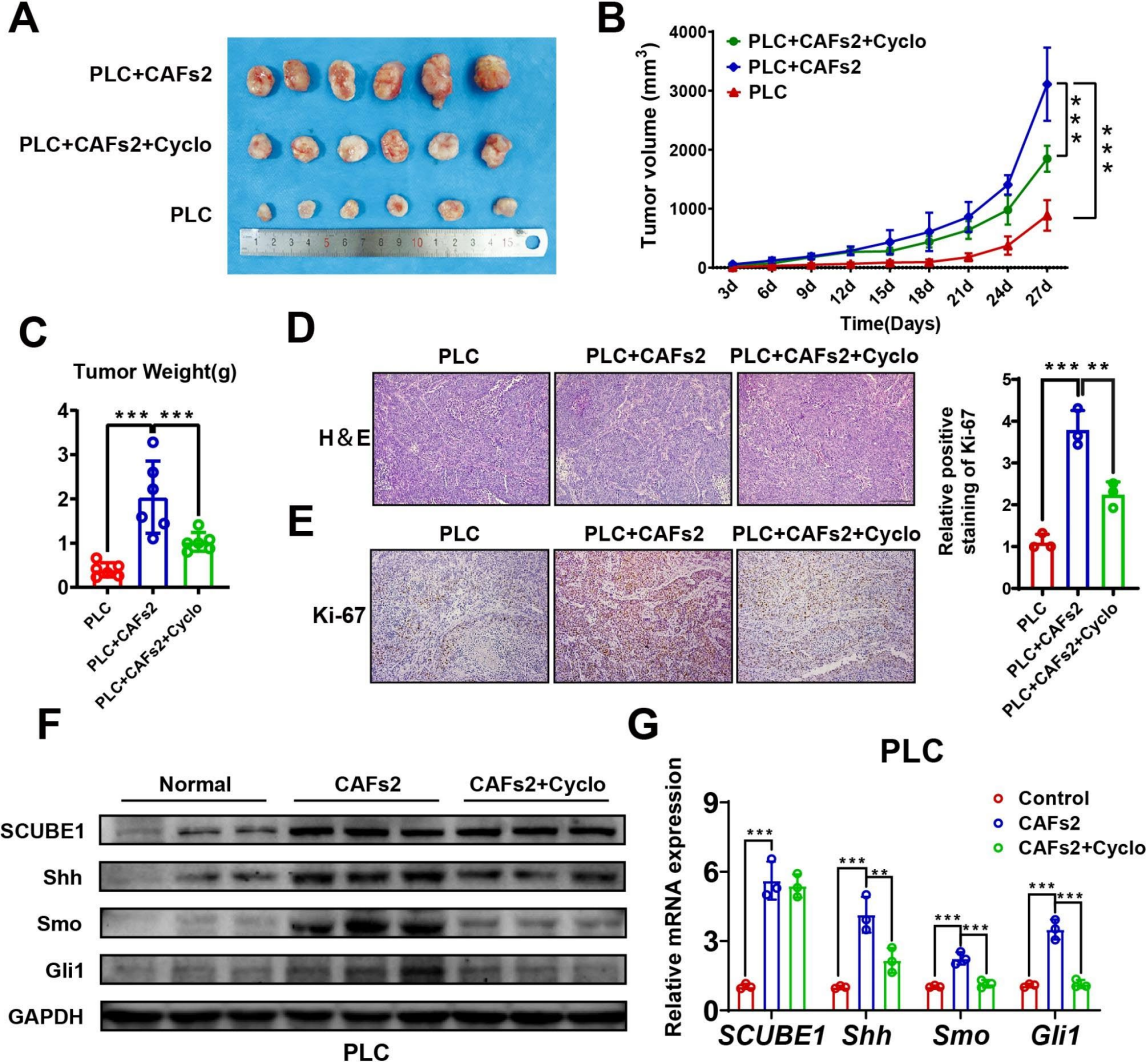
**SCUBE1 promotes tumorigenesis in vivo through the Shh pathway.(A)** Differences in tumour sizes in nude mice from different subcutaneous implantation groups. **(B)** Growth curve of mouse subcutaneous tumours derived from different treated PLC cells at the indicated time points (**P < 0.01, ***P < 0.001 vs. PLC + CAFs2, one-way ANOVA). **(C)** Differences in tumour weights in nude mice from different subcutaneous implantation groups. **(D)** Sections of HCC tissues were visualized using HE staining. Scale bar, 200μm. **(E)** Ki-67 immunohistochemical staining in different groups. Immunohistochemical staining of Ki67 expression was quantified (**P < 0.01, ***P < 0.001 vs. PLC + CAFs2, one-way ANOVA). Scale bar, 200μm. **(F)** Western blot analysis of SCUBE1, Shh, Smo and Gli1 expression in different groups was performed. GAPDH was used as an internal control. **(G)** The mRNA expression levels of SCUBE1, Shh, Smo and Gli1 in different groups were measured. β-Actin was used as an internal control (**P < 0.01, ***P < 0.001 vs. CAFs2, one-way ANOVA). Data are represented as mean ± SD of three independent experiments

Normal PLC cells and a mixture of PLC cells and CAFs2 were subcutaneously seeded into nude mice to construct xenograft tumours. When the tumour volume reached 200 mm^3^, cyclopamine was injected into nude mice at a dose of 25mg/kg per day. After two weeks, all the nude mice were sacrificed, and the tumours were collected, photographed, weighed, and stored. The tumour growth curve and tumour weight revealed that the group engrafted with both PLC cells and CAFs2 had a higher proliferation rate than the normal PLC group and the cyclopamine-treated group (Fig.6A-C). The above results indicate that CAFs2 with high SCUBE1 expression can promote the progression of HCC in vivo and that the use of cyclopamine can partially inhibit this promotion. Immunohistochemical staining of Ki-67 showed that the coculture group had the highest growth rate, while the ratio of Ki-67 in the cyclopamine-treated group was higher than that in the control group (Fig.6D-E). In addition, after the use of cyclopamine, the expression levels of Shh, Smo and Gli1 were reduced to varying degrees, but SCUBE1 was almost unaffected. (Fig.6F-G). Based on these findings, we concluded that CAFs with high SCUBE1 expression and secretion can promote the progression of HCC in vivo via the Shh pathway.

### 7. HCC patients had high SCUBE1 expression and a poor prognosis

**Fig. 7:**
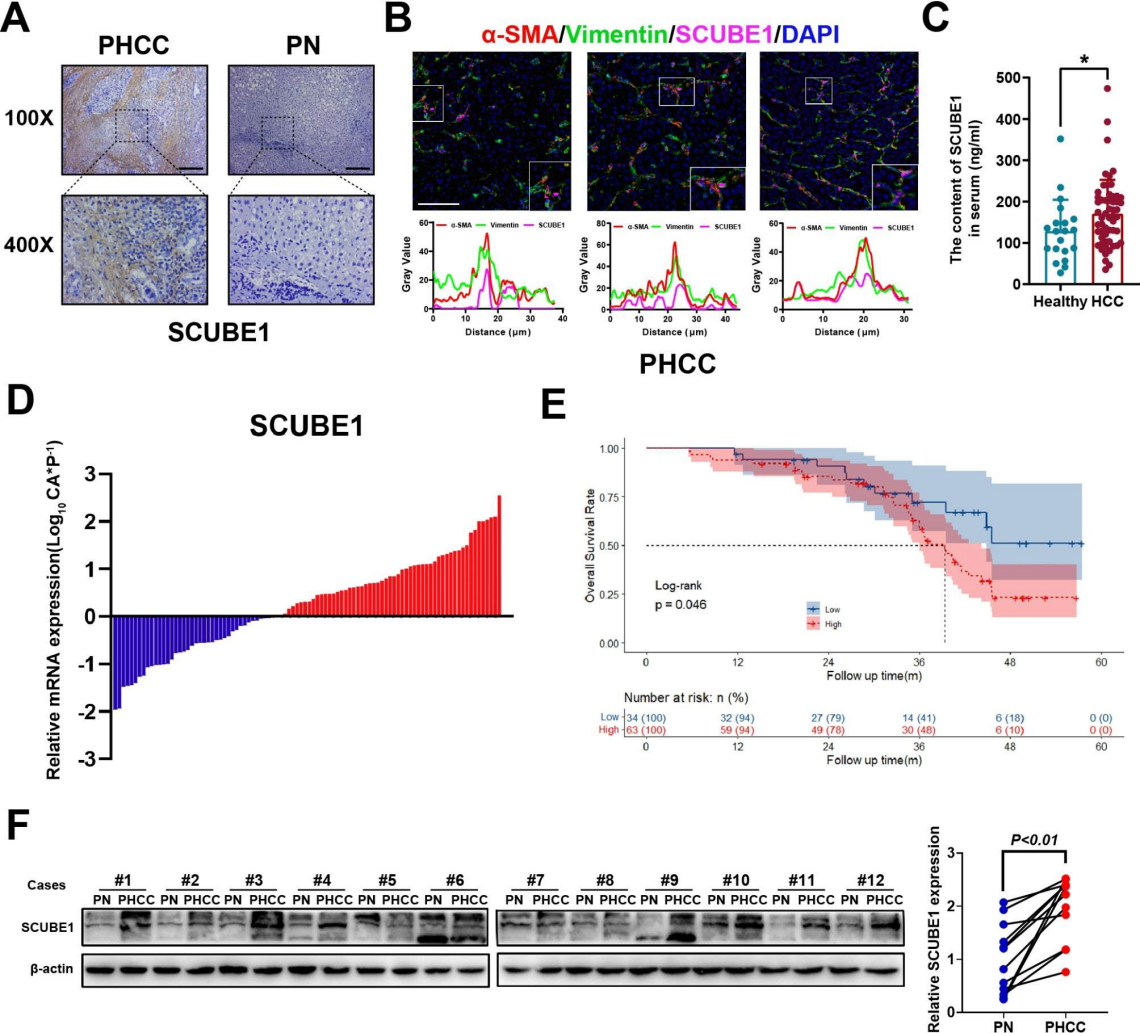
**HCC patients had high SCUBE1 expression and a poor prognosis.(A)** IHC staining for SCUBE1 was performed in HCC tissues and paracancerous tissues. Scale bar, 200μm. **(B)** HCC sections were stained with α-SMA (Red), vimentin (Green) and SCUBE1 (Cyan) by mIHC. Scale bar, 100μm. **(C)** The SCUBE1 content in the serum of HCC patients and healthy people was detected. **(D)** Relative *SCUBE1* mRNA expression was evaluated by RT–qPCR. **(E)** Survival analysis was performed using KM plotter (P = 0.046 using log-rank test). **(F)** SCUBE1 expression was detected in HCC tissues and paracancerous tissues. β-Actin was used as an internal control (*P < 0.05, t test). Data are represented as mean ± SD of three independent experiments

To clarify the expression of SCUBE1 in HCC tissues and adjacent tissues, immunohistochemical staining of SCUBE1 was first performed. According to the results of IHC, SCUBE1 was mainly expressed in the tumour stroma with higher expression in tumour tissues than that in normal tissues (Fig.7A). To observe its relative localization with CAFs marker, we performed mIHC of FFPE tissue sections and stained them with α-SMA, vimentin and SCUBE1. According to the results, it could be found that the expression of SCUBE1 varies with the changes of α-SMA and vimentin (Fig.7B). Subsequently, serum was collected from 20 healthy individuals and 56 HCC patients, and ELISAs revealed that the SCUBE1 content in the serum of HCC patients was higher than that in the serum of healthy people (Fig.7C). SCUBE1 mRNA detection via RT–qPCR in 97 patient tissues also showed high expression in most patients (57.7%) (Fig.7D). The detailed clinicopathological characteristics of the 97 participating patients with HCC are summarized in Table3. Then, KM survival analysis revealed that a high level of SCUBE1 was related to poor survival (Fig.7E). Then, WB was used to detect the expression of SCUBE1 in HCC tissues and paracancerous tissues, and SCUBE1 was found to be highly expressed in HCC tissues (Fig.7F). Overall, we can conclude that HCC tumour tissues and the circulatory system of HCC patients have high SCUBE1 expression. This finding provides new guidance and suggestions for clinical treatment.

**Table:** Table3

Pathology Character n		SCUBE1 Expression		P Chi-squared test P-value
		**Low cases(34) High cases(63)**		
**Gender**				0.059
**Female**	22	4	18	
**Male**	75	30	45	
**Age(Year)**				0.741
**<=60**	52	19	33	
**> 60**	45	15	30	
**TNM Stage**				0.048
**Stage I ~ II**	71	29	42	
**Stage III ~ IV**	26	5	21	
**Neoplasm Histologic Grade**				0.845
**G1 ~ G2**	66	22	42	
**G3 ~ G4**	33	12	21	
**BMI**				0.394
**≤ 24.9**	69	26	43	
**> 24.9**	28	8	20	
**Liver cirrhosis**				0.014
**NO**	12	8	4	
**YES**	85	26	59	
**AFP(ug/L)**				0.226
**≤ 9**	35	15	20	
**> 9**	62	19	43	
**CEA(ug/L)**				0.698
**≤ 5**	68	23	45	
**> 5**	29	11	18	
**Recurrence**				0.079
**NO**	51	22	29	
**YES**	46	12	34	
**Smoking**				0.226
**NO**	62	19	43	
**YES**	35	15	20	
**Drinking**				0.098
**NO**	62	18	44	
**YES**	35	16	19	
**Vascular invasion**				0.029
**NO**	60	26	34	
**YES**	37	8	29	

Detailed clinicopathological characteristics of 97 participated patients with HCC are summarized in Table3

## Discussion

HCC often occurs in patients with chronic liver disease. Patients with chronic liver disease usually experience hepatitis, liver fibrosis, cirrhosis, and finally, liver cancer. [25]; Studies have shown that genes in paracancerous tissues can determine the survival rate of HCC patients rather than genes in cancer tissues, which shows that the TME plays an important role in HCC development and metastasis. [26] One of the distinguishing characteristics of liver cancer is that the occurrence and development process is closely related to fibrosis, and 80–90% of liver cancers develop on the basis of a fibrotic liver or cirrhotic liver. [27] In a fibrotic liver, genes expressed by HSCs can be activated to promote carcinogenesis by differentiating stellate cells into fibroblasts with stronger proliferation, migration and contraction abilities. Static HSCs can enhance proliferation and increase the intracellular matrix content to promote the progression of liver fibrosis. [28] In the normal liver, the extracellular matrix is mainly composed of type IV and type VI collagen, but in the fibrotic liver, fibrous collagens, such as type I and type III collagens, accumulate rapidly and become the main component of the extracellular matrix, and the amount of noncollagen glycoproteins also increases significantly. [29] The deposition of the extracellular matrix can lead to a decrease in the activity of matrix metalloproteinases (MMPs), which is conducive to the formation of fibrotic scars.[30] In most liver disease studies, it has been found that the main source of α-SMA-expressing fibroblasts is HSCs.[31] Thus, we cocultured HSCs with HCC cells; this coculture system activated the HSCs, which played a role similar to that of CAFs. Activated LX2 cells were verified to promote the proliferation and stemness of HCC cells. Based on these results, gene sequencing showed that the expression of SCUBE1 in cocultured LX2 cells was significantly increased. Therefore, SCUBE1 is also likely to play a role in CAFs. CAFs are one of the most common cells in the TME and play an important role in the interaction between the tumour and tumour stroma. Studies have found that CAFs can increase the ratio of Bcl-2/BAX through the SDF-1/CXCR4/PI3K/AKT signalling pathway, thereby inhibiting the apoptosis of Huh7 cells and promoting HCC progression. [32] In addition, CAFs can oxidize fatty acids and promote glycolysis in cancer cells by upregulating CPT1A, thereby further promoting the proliferation, migration and invasion of colon cancer cells.[33] CAFs are quite different from normal fibroblasts. α-SMA, fibroblast activation protein (FAP), fibroblast specific protein 1 (FSP1), integrin β1 (CD29), platelet-derived growth factor receptor α or β (PDGFRα/β) or pudoranin (PDPN) expression can be used to distinguish CAFs from NFs. [34–37] In most experiments, α-SMA, FAP and vimentin are the most commonly used markers to identify CAFs. Therefore, we used immunofluorescence to evaluate the expression of α-SMA and vimentin in the extracted fibroblasts. The extracted fibroblasts were found to be highly positive for both α-SMA and vimentin. This also confirmed that the extracted cells were indeed CAFs.

The signal peptide-CUB-EGF domain (SCUBE) protein family contains three members with different domains, named SCUBE1, 2, and 3, and is highly associated with inflammation- and hypoxia-related diseases. [38] SCUBE1 is a secreted glycoprotein anchored on the surface of cell membranes and is mainly involved in bone development and the occurrence and development of vascular diseases. [15] Studies have shown that SCUBE1 is highly expressed in CAFs in prostate cancer, and overexpression of SCUBE1 can inhibit the cancer-promoting activity of CAFs. [39] However, studies have also reported that the concentration of SCUBE1 in the serum of patients with multiple cancer types, such as breast cancer, gastric cancer and renal cell cancer, is higher than that in healthy people. [17, 40, 41] Therefore, SCUBE1 may play different roles in promoting or inhibiting different cancers. The liver is an organ with strong regenerative capacity. It is not clear whether SCUBE1 can promote or inhibit HCC. Recent research described the heterogeneous subset of malignant hepatocytes and their functions in shaping the immune microenvironment.[42] We analyzed the TISCH and found that the expression of SCUBE1 was higher in CAFs than that in tumour cells in most cancers. Although the SCUBE1 expression levels in various cells are higher than that in tumour cells, it cannot be determined whether SCUBE1 is enriched in the tumour stroma, since the scRNA database can only assess the intracellular expression. Besides, it has not been determined in HCC. Thus, we detected SCUBE1 expression and secretion in CAFs and in HCC patients. The results showed that CAFs generally had high SCUBE1 expression and secretion and that the serum of HCC patients also had higher SCUBE1 content than that of healthy people. Besides, mIHC showed that in the region where α-SMA and vimentin were expressed together, SCUBE1 was always strongly positive. Cancer stem cells (CSCs) have self-renewal and pluripotency abilities and other stem cell properties. CSCs extracted from liver cancer are called liver cancer stem cells (LCSCs) and can promote HCC occurrence and enhance radiotherapy resistance and chemotherapy resistance.[43] LCSCs can also promote the metastasis and increase the homing ability of liver cancer cells. [44] Thus, LCSCs play a vital role in HCC tumorigenesis. Isolated LCSCs can specifically express CD133. Reducing the expression of CD133 can block the homing ability of liver cancer cells and reduce the carcinogenesis rate of HCC cells. [45] Compared with CD133-negative cells, CD133-positive cancer cells have higher proliferation, colony formation, self-renewal and differentiation abilities. [46] Increased expression of CD133 in cancer tissue samples from HCC patients is always accompanied by lower overall survival, a poorer prognosis and a higher recurrence rate. [47] In our experiments, we found that HCC cell proliferation and migration ability and CD133 expression were significantly increased after cocultivation with CAFs with high SCUBE1 expression, which showed that stemness transformation had begun in the HCC cells. Moreover, the promotive effect of SCUBE1 on the stemness transformation and malignant progression of HCC cells was also verified.

The Hh pathway plays an important role in regulating cell differentiation, the development of tissues and organs, and oncogenesis. [20] When the Hh signal is abnormally activated, Hh can promote the EMT process by activating Gli1 and increasing the malignancy of HCC cells. [48] There are three types of Hh: Sonic Hedgehog (Shh), Indian Hedgehog (Ihh) and Desert Hedgehog (Dhh). [49]Among them, Shh can directly regulate Gli1 expression. [50] Lauth et al. found that Hh signalling in lung cancer stromal cells can be activated by Shh secreted by cancer cells and that activated stromal cells can secrete a large number of cytokines to promote the malignant phenotype of lung cancer cells. [51] In addition, Hh signalling has been demonstrated to be a key factor in the tumorigenesis of breast cancer. [52] The PI3K/AKT pathway can also induce excessive activation of the Hh pathway to increase the drug resistance of breast cancer. [53] After treatment of liver cancer cells with Shh protein, the invasion and migration ability of cells can be enhanced. [54] The Shh pathway has also been shown to be an important pathway to regulate the self-renewal and maintenance of stem cells. Chen et al. reported that well-differentiated CD133(+)/ALDH(+) or CD133(+)/EpCAM(+) Huh7 and Hep3B cells showed characteristics similar to those of HCC stem cells. [55] The enhanced Hh signal transduction activity in these cancer stem cells is also one of the reasons for enhanced chemoresistance and invasion. In addition, Hh signalling can stimulate HSCs to differentiate into myofibroblasts and induce glycolysis in mesenchymal fibroblasts. [56–58] High expression of Gli1 mRNA in liver cancer tissue is negatively correlated with tumour-free survival and overall survival. [59] The SCUBE family has been verified to be a key factor in Hh signal transcription, which can promote Sonic Hedgehog (Shh) protein secretion. [18, 19, 21] In view of the above findings, we investigated the relationships between SCUBE1 and the Shh pathway and found that increased expression of SCUBE1 can promote the proliferation and migration of HCC cells by activating the Shh pathway. When this pathway was blocked, the promotive effect of SCUBE1 was significantly inhibited. This study also indicated that SCUBE1 functions by regulating the Shh pathway. Therefore, we conclude that changes in the SCUBE1 protein level can cause changes in the Shh pathway and that there is a positive correlation between SCUBE1 and Shh. In addition, the stemness and malignancy of HCC cells changes with stimulation with SCUBE1.

### Conclusion

In conclusion, SCUBE1 is highly expressed and secreted by CAFs and can promote the stemness and malignancy of HCC cells by activating the Shh/Gli1 pathway. Therefore, SCUBE1 has the potential to be a prognostic indicator of HCC patients and can be considered a target for HCC therapy. Further research is expected to reveal that SCUBE1 has an auxiliary anticancer effect.

**Supplementary Fig.1. ScRNA-seq of SCUBE1 mRNA in kinds of cancers.**

TISCH was used to analysed the SCUBE1 mRNA in different cell populations in kinds of cancers.

**Supplementary Fig.2. The effect of CAFs2 on HCC motility and the effect of silencing SCUBE1 on CAFs markers.**

(A) The effect of CAFs on the motility of PLC cells was investigated via wound healing assays (***P < 0.001 vs. control, one-way ANOVA). (B) The effect of CAFs on the motility of Huh7 cells was investigated via wound healing assays (***P < 0.001 vs. control, one-way ANOVA). (C) The effect of SCUBE1 knockdown on CAF markers was measured using WB. Data are represented as mean ± SD of three independent experiments.

**Supplementary Fig.3. The effects of rhSCUBE1 and anti-Shh antibody on the pathway and the half-maximal inhibitory concentration (IC50) of cyclopamine were measured in PLC and Huh7 cells.**

(A) Changes in Smo and Gli1 expression in PLC and Huh7 cells were detected via WB after incubation with rhSCUBE1 and anti-Shh antibodies. (B) The IC50 of cyclopamine in PLC cells was calculated. (C) The IC50 of cyclopamine in Huh7 cells was calculated. Data are represented as mean ± SD of three independent experiments.

## Electronic supplementary material

Below is the link to the electronic supplementary material.

Supplementary Material 1

Supplementary Material 2

Supplementary Material 3

## Data Availability

The datasets supporting our fndings are presented in the article.

## Abbreviations

HCC: hepatocellular carcinoma
CAFs: cancer-associated fibroblasts
TME: The tumor microenvironment
Shh: Sonic Hedgehog
HSC: Hepatic stellate cell
IHC: Immunohistochemistry
RT-qPCR: Real Time Quantitative PCR
LCSC: liver cancer stem cells
IHC: immunohistochemistry
TCGA: The Cancer Genome Atlas
