# Supplementary figures and images for: CAFs-derived SCUBE1 promotes malignancy and stemness through the Shh/Gli1 pathway in hepatocellular carcinoma
Source: J Transl Med. 2022 Nov 8;20:520. doi: 10.1186/s12967-022-03689-w (PMC9644546; doi:10.1186/s12967-022-03689-w)

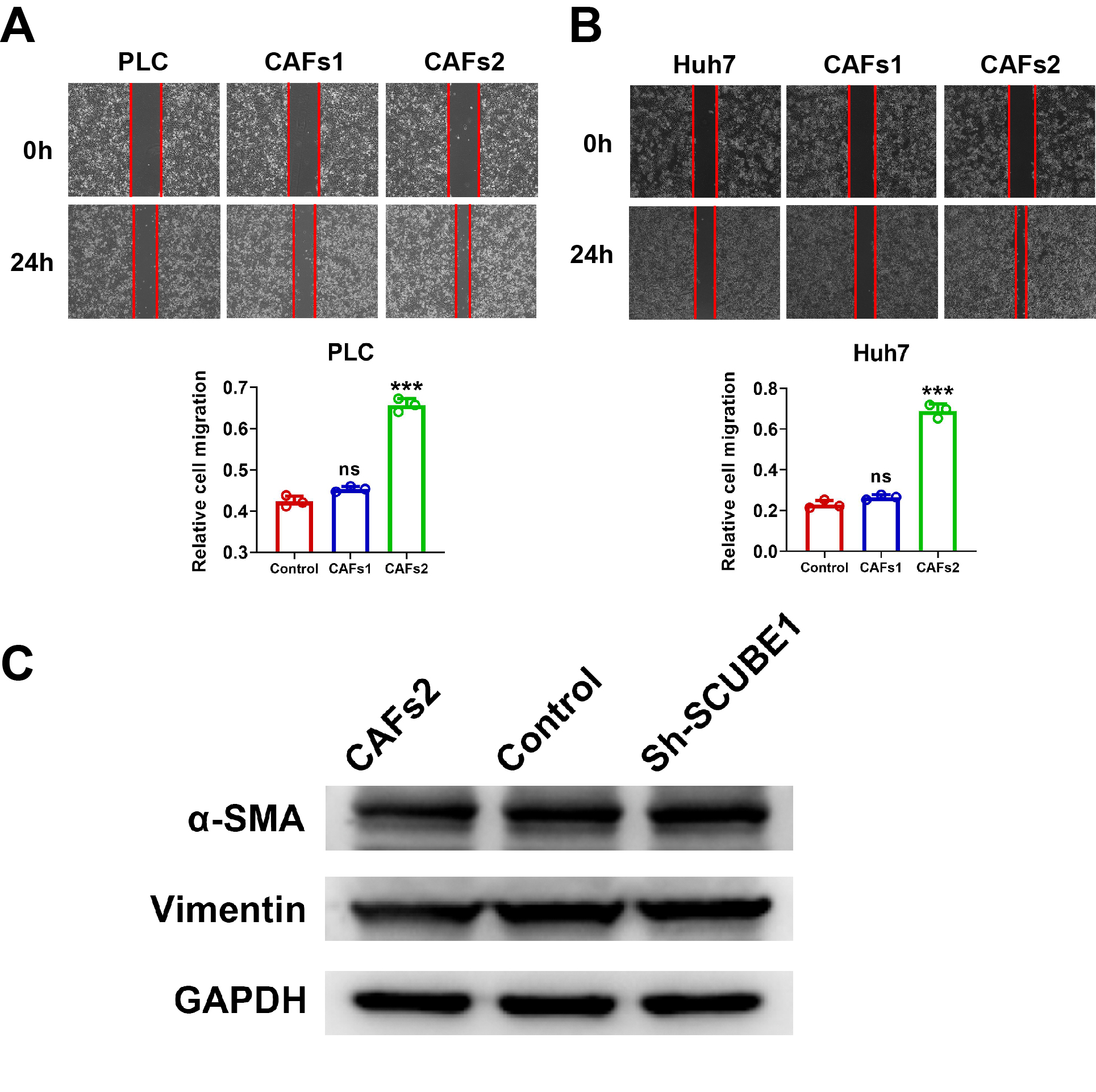

Supplement: Supplementary file 2 — Supplementary Material 2 [file 12967_2022_3689_MOESM2_ESM.jpg]

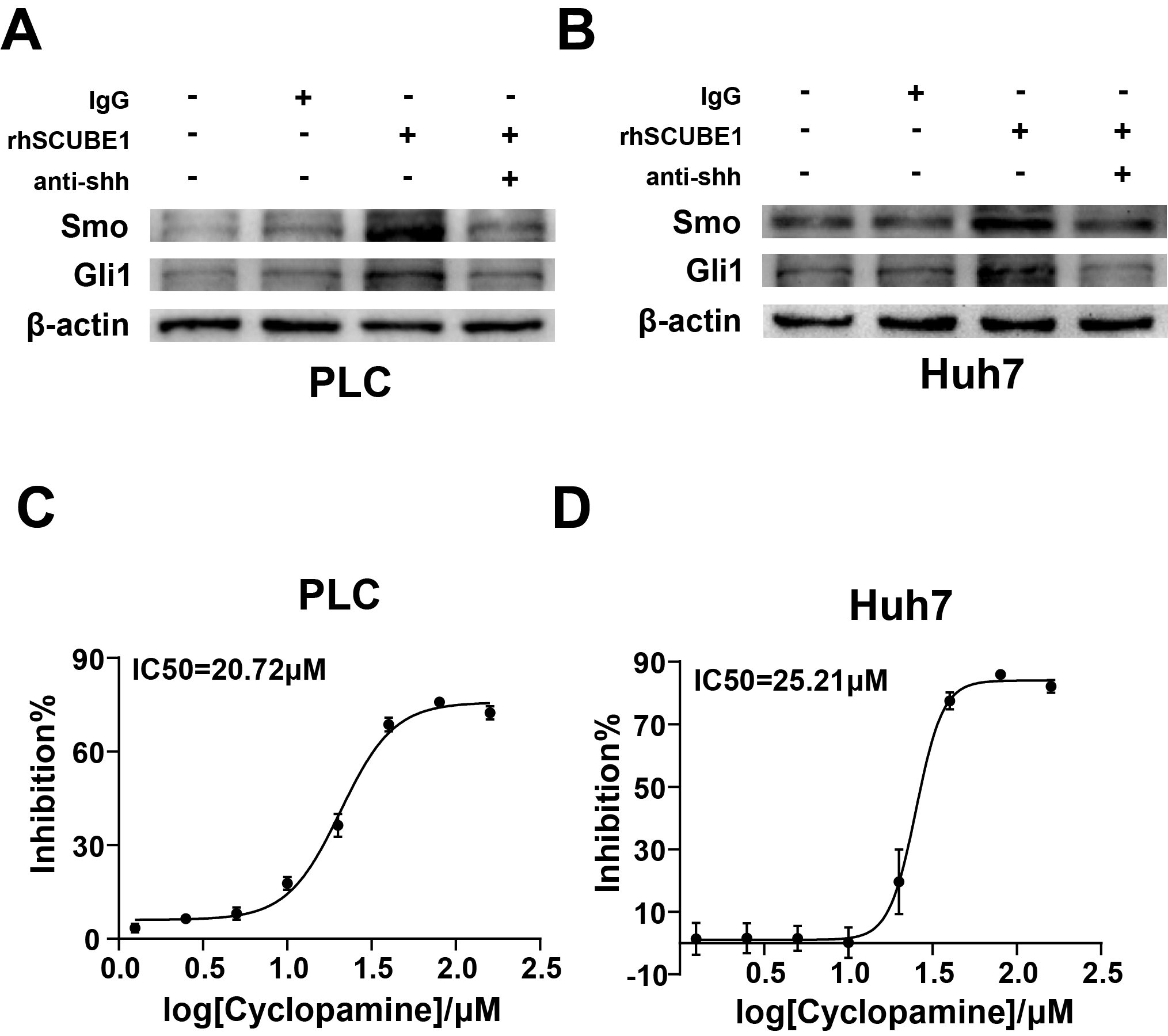

Supplement: Supplementary file 3 — Supplementary Material 3 [file 12967_2022_3689_MOESM3_ESM.jpg]
